# Triggering Cation-Induced Contraction of Cytoskeleton Networks via Microfluidics

**DOI:** 10.3389/fphy.2020.596699

**Published:** 2020-11-09

**Authors:** Shea N. Ricketts, Pawan Khanal, Michael J. Rust, Moumita Das, Jennifer L. Ross, Rae M. Robertson-Anderson

**Affiliations:** 1 Department of Physics and Biophysics, University of San Diego, San Diego, CA, United States; 2 Department of Molecular Genetics and Cell Biology, University of Chicago, Chicago, IL, United States; 3 School of Physics and Astronomy, Rochester Institute of Technology, Rochester, NY, United States; 4 Department of Physics, Syracuse University, Syracuse, NY, United States

**Keywords:** cytoskeleton, microfluidics, actin, microtubules, microscopy

## Abstract

The dynamic morphology and mechanics of the cytoskeleton is determined by interacting networks of semiflexible actin filaments and rigid microtubules. Active rearrangement of networks of actin and microtubules can not only be driven by motor proteins but by changes to ionic conditions. For example, high concentrations of multivalent ions can induce bundling and crosslinking of both filaments. Yet, how cytoskeleton networks respond in real-time to changing ion concentrations, and how actin-microtubule interactions impact network response to these changing conditions remains unknown. Here, we use microfluidic perfusion chambers and two-color confocal fluorescence microscopy to show that increasing magnesium ions trigger contraction of both actin and actin-microtubule networks. Specifically, we use microfluidics to vary the Mg^2+^ concentration between 2 and 20 mM while simultaneously visualizing the triggered changes to the overall network size. We find that as Mg^2+^ concentration increases both actin and actin-microtubule networks undergo bulk contraction, which we measure as the shrinking width of each network. However, surprisingly, lowering the Mg^2+^concentration back to 2 mM does not stop or reverse the contraction but rather causes both networks to contract further. Further, actin networks begin to contract at lower Mg^2+^ concentrations and shorter times than actin-microtubule networks. In fact, actin-microtubule networks only undergo substantial contraction once the Mg^2+^ concentration begins to lower from 20 mM back to 2 mM. Our intriguing findings shed new light on how varying environmental conditions can dynamically tune the morphology of cytoskeleton networks and trigger active contraction without the use of motor proteins.

## INTRODUCTION

The cytoskeleton, a dynamic network of filamentous proteins, enables cells to maintain shape and structure while carrying out a wide range of processes such as cell proliferation, migration and division. To enable such diverse processes and structural properties, cytoskeletal networks readily rearrange in response to changing environmental conditions (ions, nucleotide-triphosphates, and crowding) and interactions with accessory proteins. Two of the principle constituents of the cytoskeleton are thin semiflexible actin filaments, ~7 nm wide with a persistence length of *l*_*p*_ ~ 10 *μ*m, and thicker rigid microtubules, ~25 nm wide with *l*_*p*_ ~ 1 mm [1, 2]. Both filaments are also polyelectrolytes with an average linear charge density of 4 *e*/nm for actin filaments and 280 *e*/*μ*m for microtubules [3–5]. Actin filaments serve primary roles in migratory processes, cellular contraction and maintaining cellular polarity while microtubules organize and maintain axonal branching, intracellular trafficking and mitotic spindle orientation during cellular division [6–10]. Further, interactions between actin and microtubules play critical roles in essential dynamic processes including directed cell migration, neuronal growth, cellular wound healing, cortical flow, and cellular division [11–16].

Active reorganization of cytoskeleton networks is typically driven by ATP-consuming motor proteins including myosin and kinesin. *In vitro* actin-myosin networks are shown to be contractile, while purified microtubule-kinesin networks form extensile liquid crystal networks [17–19]. However, due to the polyelectrolyte nature of both actin and microtubules, changes to the ionic conditions of the network environment can also trigger rearrangements in actin and microtubule networks. For example, high concentrations of Mg^2+^ ions have been shown to induce bundling and crosslinking of actin filaments [20–23] and microtubules [24, 25] via counterion crossbridges. Increasing Mg^2+^ concentration has also been shown to promote higher order structure formation in both actin and microtubule networks *in vitro* and *in vivo* [26–31].

We previously showed that the mechanical response of actin networks depended strongly on the Mg^2+^ concentration. Specifically, actin networks polymerized at Mg^2+^ concentrations of 2–52 mM exhibited an increase in network stiffness, nonlinear force response, elasticity and relaxation timescales with increasing Mg^2+^ concentration. We showed that this increase in the mechanical response arose from small-scale counterion-enabled crosslinking and bundling of actin filaments that occurred for Mg^2+^ concentrations ≥10 mM Mg^2+^. Surprisingly, despite dramatic changes in mechanical behavior, the mesoscopic changes to network morphology and architecture were relatively small in nature [22].

While varying Mg^2+^ concentration has been shown to impact both actin and microtubules, no studies have investigated the effects of Mg^2+^ on composite actin-microtubule networks. Moreover, how dense cytoskeleton networks dynamically morph from one state to another as ion concentration changes remains unknown. To address these open questions, we use microfluidic perfusion chambers to slowly vary the Mg^2+^ concentration between 2 and 20 mM while imaging the triggered changes in actin and actin-microtubule networks using two-color confocal microscopy. Specifically, we characterize how the bulk network size changes as we vary the Mg^2+^ concentration from low (2 mM) to high (20 mM) and back to low (2 mM). We show that increasing the Mg^2+^ concentration triggers bulk contraction of both actin networks and actin-microtubule networks. However, when subsequently lowering the concentration from 20 to 2 mM Mg^2+^ both actin and actin-microtubule networks surprisingly continue contracting rather than re-expanding or stopping contraction. Moreover, as we describe here within, while both networks contract, there are marked differences in the contraction rate and characteristics for actin vs. actin-microtubule networks.

## METHODS

### Sample Preparation

**FIGURE 1 |:**
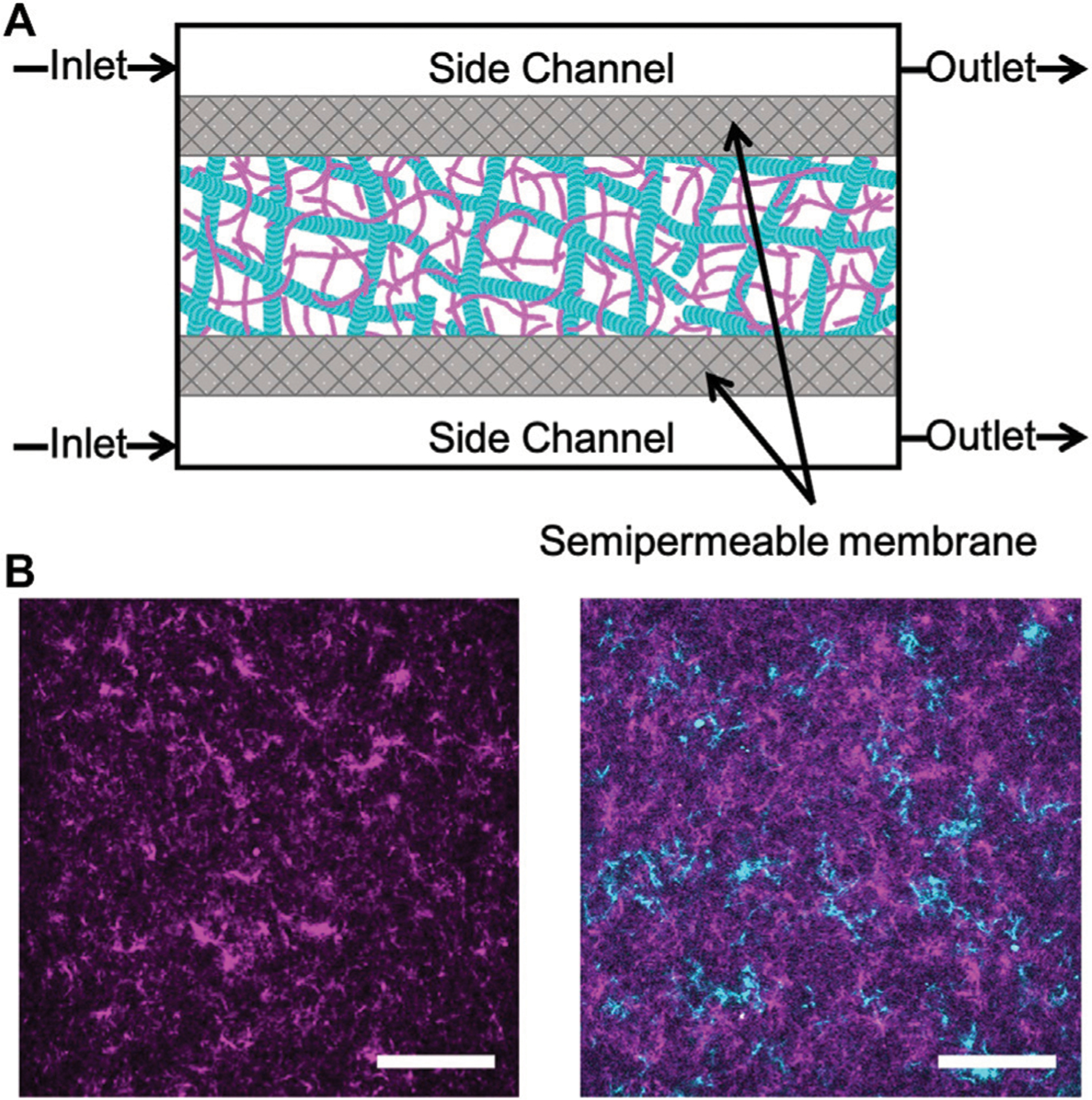
Experimental approach to triggering cation-induced contraction of cytoskeleton networks. **(A)** Cartoon of microfluidic device comprised of three channels separated by two semipermeable membranes (gray). The device has a central chamber containing the sample and two side channels used for buffer exchange. Buffer is pulled into the inlet and out of the outlet through capillary tubing using a syringe pump. The flow rate is set to allow for passive diffusion of buffer into the sample chamber as it flows from inlet to outlet. **(B)** Two-color laser scanning confocal images of an actin network **(left)** and an equimolar actin-microtubule network **(right)** where actin is magenta and microtubules are cyan. Scale bar is 50 *μ*m.

Rabbit skeletal actin and Alexa-568-labeled actin were purchased from Cytoskeleton (AKL99) and Thermofisher (A12374) and stored at −80°C in Ca buffer [2 mM Tris (pH 8.0), 0.2 mM ATP, 0.5 mM DTT, 0.1 mM CaCl_2_]. Porcine brain tubulin and rhodamine-labeled tubulin were purchased from Cytoskeleton (T240, TL590M) and stored at −80°C in PEM-100 (100 mM PIPES (pH 6.8), 2 mM MgCl_2_, and 2 mM EGTA). Both actin and actin-microtubule networks were formed at a fixed concentration of 5.8 *μ*M total protein concentration and included a fraction of labeled protein to image networks (Figure 1). Actin networks were polymerized from 3.9 *μ*M unlabeled actin monomers and 2 *μ*M labeled actin monomers in PEM-100 with 2 mM ATP. Equimolar actin-microtubule networks were prepared by polymerizing 2.4 *μ*M actin monomers, 0.5 *μ*M labeled actin monomers, 2 *μ*M tubulin dimers and 0.9 *μ*M labeled tubulin dimers in PEM-100 with 2 mM ATP, 2 mM GTP, and 5 μM Taxol. By including a small fraction of labeled monomers in the solution prior to network polymerization, rather than doping in pre-formed labeled filaments, we are able to directly visualize the network structure and morphology rather than relying on tracer filaments to report the structure [32]. However, the caveat to this technique is that individual filaments cannot be resolved as each filament is too sparsely labeled and the network is too dense (<1 μm mesh size). As such, the networks in Figure 1 show structure but not individual filaments. To reduce photobleaching, oxygen scavenging agents (4.5 *μ*g/ml glucose, 0.005% β-mercaptoethanol, 4.3 *μ*g/ml glucose oxidase, 0.7 *μ*g/ml catalase) were included. For both network types, the final solution was mixed and pipetted into the central channel of the microfluidic sample chamber (Figure 1) and incubated at 37°C for 30 min prior to further microfluidic assembly (described below). We have shown these networks to be isotropic and stable for up to ~48 h [33]. As such, we do not expect filament orientation or sample aging to play a role in our results.

### Microfluidics

The construction of the microfluidic device shown in Figure 1 is adapted from techniques in Ref. 34 and Ref. 35. The microfluidic sample chamber is formed from a coverslip, a glass microscope slide, and parafilm. The coverslip and glass slide were washed thoroughly with deionized water, acetone, and isopropanol and then plasma cleaned for 30 min. A mixture of 0.5% TMSPMA in isopropanol was baked onto the coverslip and glass slide at 80°C for 2 h. The slide and coverslip were washed with isopropanol and ethanol and left to air dry. To assemble the sample chamber and create a flow cell, the slide, coverslip, and parafilm spacer were placed on a 60°C plate to allow the parafilm to melt and fuse the slide and coverslip together.

The flow cell was filled with a 50:1 mixture of polyethylene glycodiacrylate (PEG-DA) and photoinitiator, 2-hydroxy-2-methylpropiophenone, diluted to 10% in 10 mM Tris-HCl, then exposed to UV through a custom photomask to form two semipermeable membranes of crosslinked PEG-DA. The flow cell was immediately flushed with DI to remove the un-linked PEG-DA solution. This process results in three channels separated by two semipermeable membranes: one central channel for holding the sample and two side channels to enable buffer exchange via diffusion. The flow cell was flushed with 5% Tween (in PEM-100) followed by PEM-100.

The sample was pipetted into the central channel and the side channels were filled with the original polymerization buffer consisting of PEM-100, 2 mM ATP and 2 mM GTP (only for actin-microtubule networks). The flanking side channels were then connected to capillary tubing (74/95 mm inner/outer diameter, Incom) at both ends. The sides of the device were sealed with epoxy to completely enclose the sample within the central chamber and the capillary tubing in the side channels. The capillary tubes were connected to separate Tygon tubing (Cole Parmer Tygon tubing AAD0209-CP, 0.010/0.030 inches inner/outer diameter) before sealing all tubing with epoxy. The Tygon tubing on the inlet side of the microfluidic device was prefilled with the original polymerization buffer. We included 2 *μ*M fluorescein salt in the original polymerization buffer as a proxy to measure ion concentration. To enable buffer exchange, the outlet Tygon tubes were connected to a digitally controlled syringe pump and the inlet Tygon tubing was inserted into the desired buffer reservoir. When the syringe pump is turned on, buffer is pulled into the side channels from the reservoir at a flow rate of 3 *μ*l/min to enable buffer exchange within the sample chamber via passive diffusion through the semipermeable membranes.

The microfluidic experiment took place over the course of 3 h. In the first 10 min, the pump remained off in order to equilibrate the system. We then pumped in the fluorescein-polymerization buffer for 30 min to achieve simultaneous flow at an equal flow rate. After 30 min of pumping the fluorescein-polymerization buffer we switched the reservoir to the buffer containing PEM-100, 20 mM MgCl_2_, 2 mM ATP and 2 mM GTP (only for actin-microtubule networks). This buffer did not contain fluorescein. We pumped this buffer through for 90 min before switching back to the fluorescein-polymerization buffer and pumping for 50 min.

### Confocal Imaging

A Nikon A1R laser scanning confocal microscope with a 4× objective and QImaging QICAM CCD camera was used to collect time-series of labeled cytoskeletal networks and buffer channels. The low magnification objective enabled us to visualize the entire network to determine bulk morphological changes triggered by buffer exchange. It also allowed us to view the entire device in a single field of view to characterize the rate of buffer exchange and to correlate the network activity with Mg^2+^ concentration. The microscope is outfitted with 488 nm and 561 nm lasers and simultaneously records separate images for each laser channel. As such, we are able to separately visualize the cytoskeleton network in the sample chamber (568 nm laser channel) and the intensity of fluorescein dye in the side channels and sample chamber (488 nm laser channel). Time-series of 512 × 512 images (6.215 *μ*m/pixel) in each channel were recorded for 3 h with each image taken every minute. Time-series from the network channel and fluorescein channel were analyzed separately using custom written Matlab code to determine network width and corresponding Mg^2+^ concentration.

## RESULTS

We use microfluidic perfusion chambers and two-color confocal microscopy to measure the bulk structural changes induced in cytoskeletal networks in response to continuous variation of Mg^2+^ concentration (Figure 1, Methods). We prepare entangled actin networks and co-entangled actin-microtubule networks using previously established protocols [32, 33]. We polymerize networks within home-built microfluidic chambers to enable subsequent variation of Mg^2+^ concentration, via passive diffusion, without disrupting the network of polymerized filaments.

**FIGURE 2 |:**
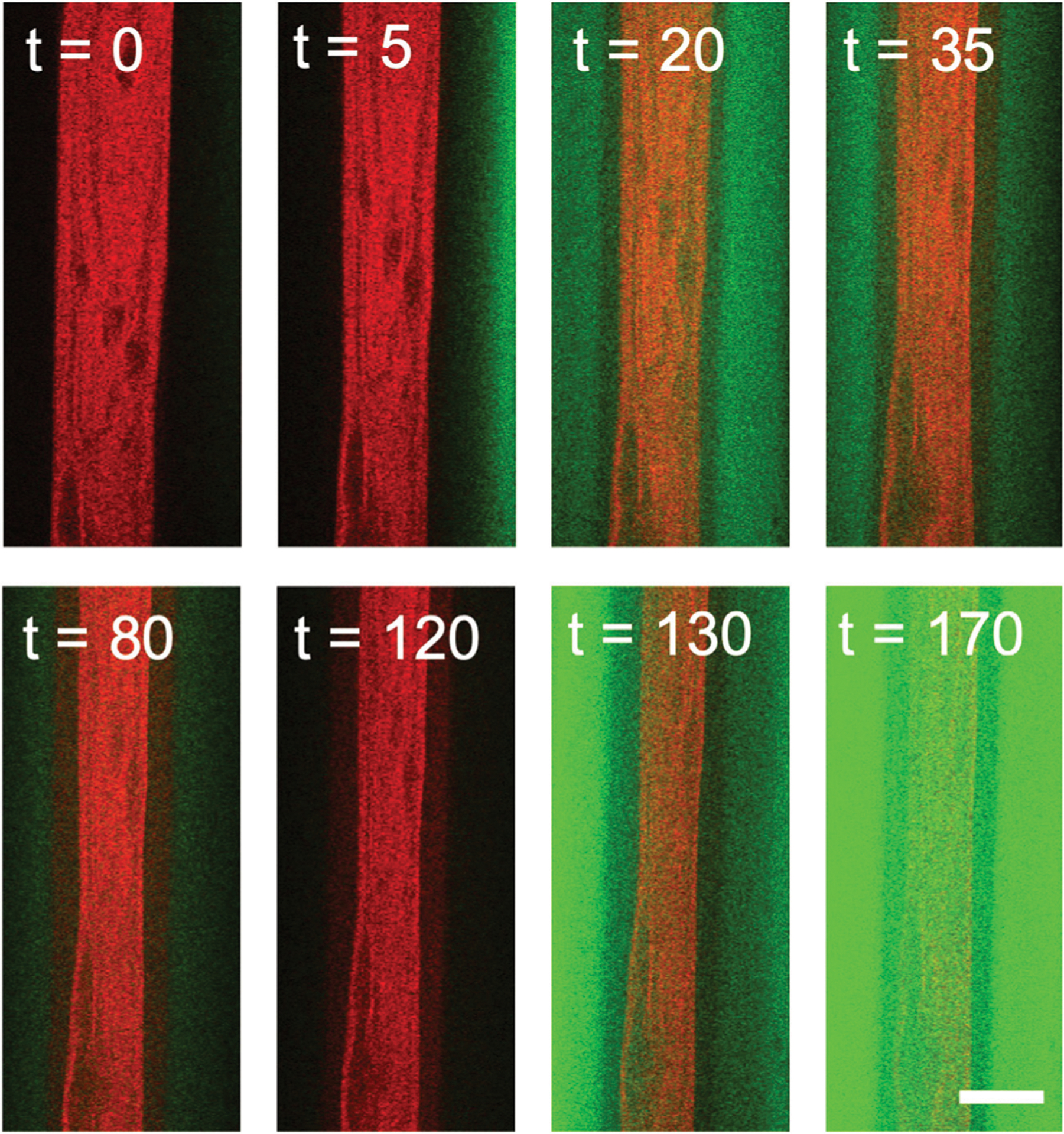
Two-color confocal imaging of actin network undergoing contraction triggered by variation in Mg^2+^ concentration. Two-color laser scanning confocal images of an actin network (red) as the Mg^2+^ concentration slowly varies from 2 mM (green) to 20 mM (black) and back to 2 mM (green). Fluorescein in the 2 mM Mg^2+^ buffer (but not in the 20 mM Mg^2+^ buffer) is used to quantify the Mg^2+^ concentration as a function of time. Because it takes ~5 min for the buffer from the reservoir to enter the sample channel, the buffer channels in the first few images are black despite being at 2 mM Mg^2+^. At t = 30 min, the 20 mM Mg^2+^ solution is introduced, which is seen as the green signal intensity decaying to black. At t = 120 min, the 2 mM Mg^2+^ solution is reintroduced, viewed as increasing intensity in the green channel. Scale bar is 500 *μ*m and time is in minutes.

Once networks are formed in microfluidic chambers, each 170 min experiment proceeds as follows (depicted in Figure 2). For the first 30 min, we pump the original 2 mM Mg^2+^ polymerization buffer through the device, allowing for passive diffusion of the buffer into the network (Phase I). This phase serves as a control to ensure that we achieve simultaneous flow with equal flow rate through both side channels. After 30 min we change the buffer to include 20 mM Mg^2+^ (Phase II). After 90 min of 20 mM Mg^2+^ buffer flow (t = 120 min), we reintroduce the original 2 mM Mg^2+^ polymerization buffer (Phase III).

**FIGURE 3 |:**
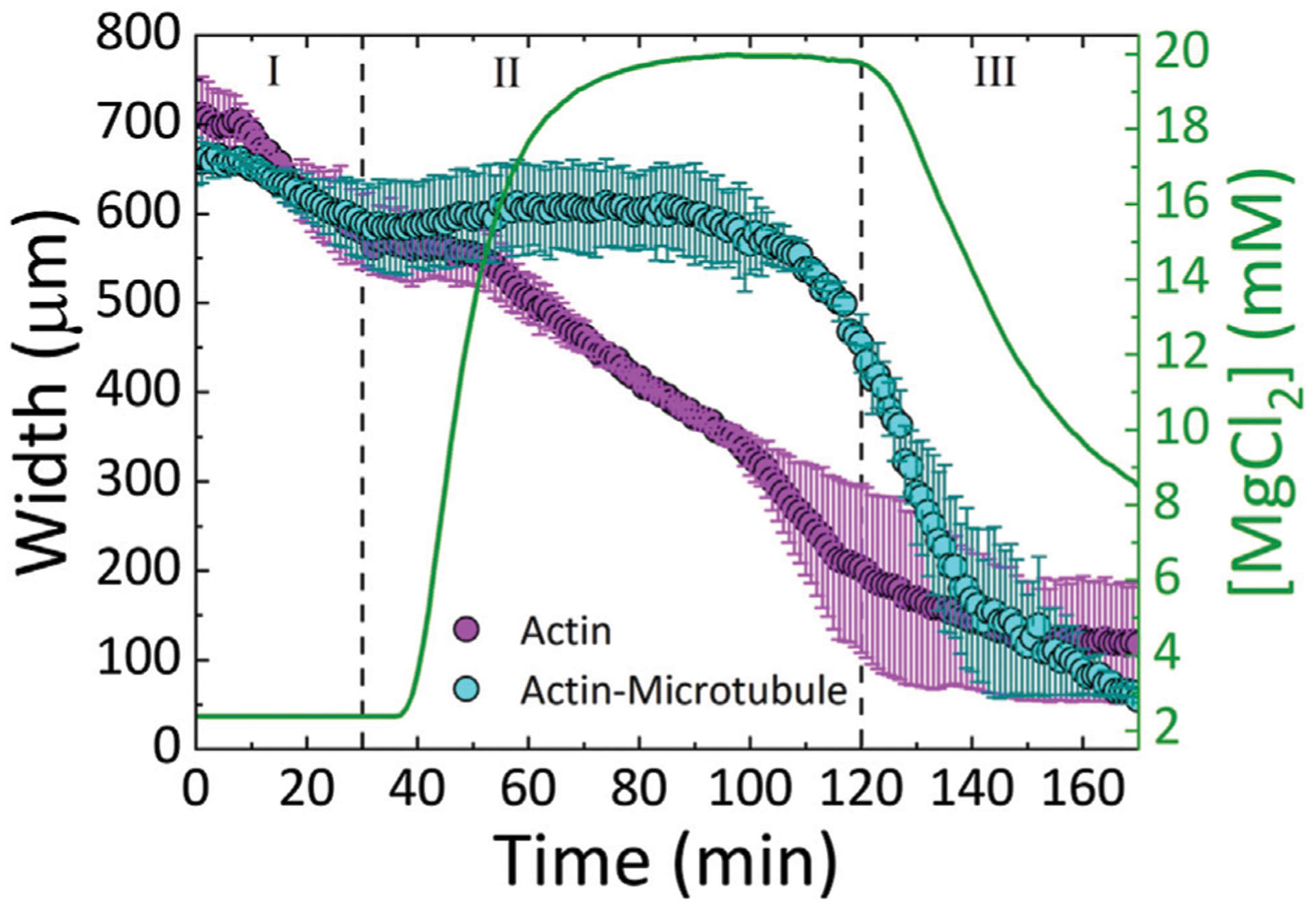
Actin and actin-microtubule networks contract in response to continuous variation of Mg^2+^ concentration. The average width (left axis) of the actin network samples (magenta) and actin-microtubule network samples (cyan) as a function of time. The Mg^2+^ concentration (right axis) is also plotted (green line) as a function of time. The three phases of the experiment (I-III) are separated by dashed lines: I) 2 mM Mg^2+^ solution (original polymerization buffer) diffuses through the sample for 30 min II) exchange to 20 mM Mg^2+^ buffer is initiated and proceeds until 120 min III) 2 mM Mg^2+^ buffer is reintroduced for 50 min.

Because buffer exchange occurs via passive diffusion, the ion concentration in the network sample chamber does not instantaneously change when we switch buffers. Thus, to determine the Mg^2+^ concentration as a function of time during the experiment we include fluorescein dye in the 2 mM Mg^2+^ buffer. We determine the relative ion concentration, which increases from 2 to 20 mM, by monitoring the decay of the fluorescein intensity. When we reintroduce the 2 mM Mg^2+^ buffer we use the fluorescein intensity once again to measure ion concentration as it decreases from 20 mM to 2 mM Mg^2+^ (Figures 2, 3). We simultaneously image the cytoskeleton network as shown in Figure 2.

**FIGURE 4 |:**
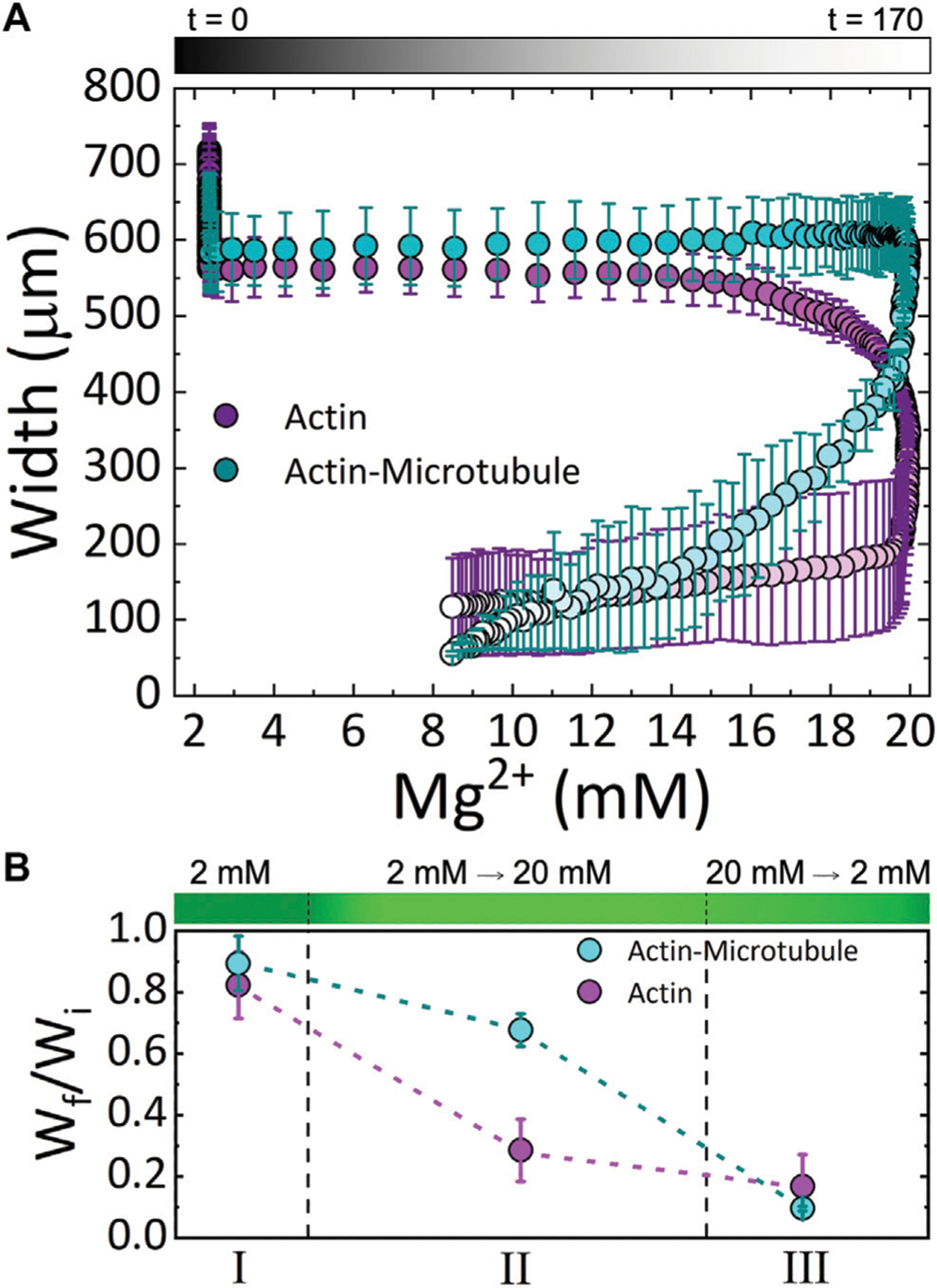
Cytoskeleton networks exhibit network-dependent contraction in response to both increasing and decreasing Mg^2+^ concentration **(A)** The average width of actin network samples (magenta) and actin-microtubule network samples (cyan) as a function of Mg^2+^ concentration. Experimental time is indicated in the symbol gradient coloring where t = 0 is cyan and magenta and t = 170 is light cyan and magenta. **(B)** The fractional amount of contraction measured for each Phase (I, II, III; described in the text), computed by W_f_/W_i_ where W_i_ is the width at the beginning of the experiment and W_f_ is the width at the end of the corresponding Phase. The dashed lines separating the three Phases approximate the relative length of time of each phase. The corresponding Mg^2+^ concentration is depicted as a gradient with dark to light green indicating 2 to 20 mM Mg^2+^.

We find that both actin and actin-microtubule networks contract as the ion concentration increases, shown by the shrinking width of the material over the course of the experiment (Figures 2–4). The extent of contraction over the full course of the experiment is similar for both networks, with both widths shrinking to ~13% of their initial width (Figure 4).

Due to the ability to control the concentration of the ions with the perfusion chamber, we can identify three phases of ion concentration resulting in changes in the network organization. Phase I is the time when buffer is flowed through the chamber, but the ionic strength of the buffer is constant. Phase II is the time frame when we increase the ion concentration. Phase III is when we return to the original buffer conditions.

As shown in Figure 3, during Phase I, the widths of both actin and actin-microtubule networks remain fairly stable, as expected as the environmental conditions are not changing significantly. The only difference between the polymerization buffer pumped in vs. the polymerization buffer the networks were formed in is the lack of the oxygen scavenging system and Taxol.

In Phase II, as Mg^2+^ concentration increases from 2 to 20 mM, actin and actin-microtubule networks both contract but the contraction dynamics are markedly different for the two network types. Actin networks contract at a nearly constant rate of ~4 *μ*m/min, reaching 28 ± 10% of their initial width (Figure 3). Conversely, the width of actin-microtubule networks remains stable for the majority of this phase, only starting to contract after ~70 min of the 90 min phase, and reaching 68 ± 5% of the initial width (Figures 3, 4).

In Phase III, when we reintroduce the original 2 mM Mg^2+^ buffer, we surprisingly find that both networks continue to contract rather than re-expand or stabilize. The rate of contraction for the actin network is slowed appreciably during this phase and appears to be approaching a steady-state width, contracting by only 11% during this phase (Figures 3, 4B). However, the rate of contraction for the actin-microtubule network actually increases during this phase, dropping by 88% at a nearly constant contraction rate of ~7 *μ*m/min over the course of the 50 min phase (t = 120 min to t = 170 min, Figure 3).

These results can be seen more readily in Figure 4A in which we plot the width for both networks as a function of Mg^2+^ concentration. The color gradient indicates the experimental time that correlates with the given Mg^2+^ concentration. As shown, actin networks begin to noticeably contract when the Mg^2+^ concentration reaches ~12 mM. Noticeable contraction of actin networks continues as the Mg^2+^ concentration increases to 20 mM, with the most dramatic contraction happening between ~18 and 20 mM Mg^2+^. Conversely, actin-microtubule networks appear to remain relatively stable as Mg^2+^ concentration increases to ~20 mM, yet undergo dramatic contraction as the concentration drops from 20 to ~17 mM. While the contraction of actin-microtubule networks slows as the Mg^2+^ concentration is lowered further, they continue to exhibit more substantial contraction than actin networks during this phase.

Figure 4B summarizes our findings for the three Phases of our experiment and highlights the key results: 1) both actin networks and actin-microtubule networks undergo significant bulk contraction in response to increasing Mg^2+^ concentration; 2) both networks continue to contract even as the Mg^2+^ concentration is lowered back to the original concentration; and 3) the onset of contraction is delayed for actin-microtubule networks in comparison to actin networks.

We note that in both networks the main period of contraction, in which a constant negative slope is observed in Figure 3, exhibits the smallest standard deviation among different samples (Figures 3, 4). This small error demonstrates the reproducibility of Mg^2+^-driven contraction. The regions with larger standard deviation, at the beginning and end of the experiments, represent variations in the initial and final widths of the different samples, likely arising from small differences in the semipermeable membranes of each microfluidic chamber (Figure 1). Slight differences in membrane thickness and pore size impact the width of the central sample channel and the rate of buffer exchange, which in turn alter the initial and final network widths.

## DISCUSSION

Previous studies have shown that the charge screening from divalent Mg^2+^ cations are sufficient to enable bundling, crosslinking and reorientation of actin and microtubules *in vitro* and *in vivo* [26, 36, 37]. The onset of contraction for actin networks, occurring at ~12 mM Mg^2+^ (Figure 4), is consistent with the previously shown critical concentration of ~10 mM Mg^2+^ needed to induce actin bundling and crosslinking [21, 22]. As such, our results suggest that this self-association can trigger bulk contraction of dense cytoskeleton networks. If the networks are highly entangled, as they are here, as each filament begins to associate with its nearest neighbor the filament pulls with it other surrounding filaments that it is entangled with, resulting in overall contraction of a fully connected network.

We can also understand this contraction process by considering the entropic cost of trapping the Mg^2+^ cations between neighboring filaments [38, 39]. At low Mg^2+^ concentrations, the cations between two filaments are in an energetically favorable state (near negatively charged surfaces), and the filaments repel each other due to the cloud of positive ions surrounding them (and repelling each other). Squeezing the two filaments together would lower the entropy of the “trapped” ions and is thus avoided. This effect is similar to an osmotic pressure difference that drives water between the filaments to try to balance the concentration of cations near and far from filament surfaces [38, 40, 41]. However, as the cation concentration increases this osmotic pressure difference is lowered and then reversed as the concentration of cations in bulk increases. Thus, water flows out of the region between filaments into the bulk, driving the filaments together. The net result is overall network contraction as the cation concentration increases. It is important to note that this depletion-like effect is driven by thermal fluctuations of the filaments. Namely, thermal fluctuations that drive the filaments together, and thus push the water out, are preferred. As such, the timescale for this contraction process depends on the relaxation timescales of the filaments.

The more counterintuitive and surprising result is that contraction continues when we lower the Mg^2+^ concentration by reintroducing the original 2 mM Mg^2+^ polymerization buffer (Phase III). While Phase II contraction is largely driven by the free energy minimization of the cations, as we describe above, we interpret the Phase III contraction as arising from the free energy minimization of the filamentous network. Namely, as filaments are pulled toward each other and linked together by cation crossbridges, the initial configuration is not the most entropically favorable in terms of their configuration. There are likely mechanical stresses on the filaments that were pulled together by the increasing ion concentration. Thus, the filaments will rearrange and reorient to relieve this stress and increase their configurational entropy, even when the cation concentration is reduced. Because the relaxation timescales of entangled actin networks such as these can be as long as minutes to hours, [33, 42–44] this can indeed be a slow process. The fact that this process leads to contraction rather than re-expansion suggests that the cation crossbridges that form between filaments at high Mg^2+^ concentration are quite strong. Further, there is no obvious driving force, analogous to the entropic force that drives the filaments together, that would force the filaments apart. Finally, there are theoretical predictions that suggest that thermal Casimir forces may play a role in electrolyte solutions confined by surfaces (which in this case are the filaments) and in biopolymer networks [45, 46]. This force, arising from thermal fluctuations in the ionic concentration near filaments leads to an attractive force between filaments, similar to the van der Waals interaction [47]. This effect may also play a role in the Phase III contraction.

The question remains as to why the actin-microtubule networks exhibit contraction dynamics that are so distinct from actin networks. Namely, actin-microtubule networks require more Mg^2+^ cations (~20 mM Mg^2+^) for the onset of contraction than actin networks, and the most pronounced contraction occurs only after the ion concentration starts to drop (Figure 4). Microtubules are much stiffer than actin, with a ~100× larger persistence length, and are also comprised of more proteins per unit length. The result then of replacing half of the molar protein concentration of the actin network with tubulin is that the network is stiffer and the mesh size of the network is ~2× larger [33]. In addition, the relaxation dynamics of the actin-microtubule network are slower as the microtubules relax and reorient over much longer timescales than actin [33].

The longer relaxation times could explain the delay in contraction triggered by increasing Mg^2+^ concentration due to the time needed for the actin-microtubule network to rearrange and reorient in response to the triggered attraction. The increased mesh size of the actin-microtubule network could also delay the depletion-like self-association of filaments. As the spacing between filaments trapping the cations is larger, more thermal fluctuations (and thus longer time) and/or a stronger entropic force (dictated by the cation concentration difference between the bulk and in between filaments) would be required for neighboring filaments to be driven together by the osmotic-like depletion of water between the filaments. As such, a higher Mg^2+^ concentration and longer time would be needed to induce contraction, just as we see in Figures 3 and 4. Just as the actin-microtubule network is harder to start contracting, the composite would also be more difficult to stop contracting, as if it had an inertial response to the ions. Thus, we see a delay in the contraction, and the most significant contraction only occurring once the ion concentration begins to drop (Figure 4). Interestingly, while the contraction is delayed in actin-microtubule composites compared to actin networks, once contraction begins the rate is ~2× faster than for actin networks. This faster contraction, is most likely due to their larger mesh size. While the composites require higher Mg^2+^ concentration and more time for thermal fluctuations to move neighboring filaments close enough to each other to allow for depletion-driven contraction, once the filaments reach this point they can move together more rapidly because there are fewer steric constraints (entanglements) restricting their motion. Namely, the empty voids in the mesh that filaments can freely move through are larger than in actin networks. While actin filaments are more flexible and can thus more readily respond to depletion forces, their smaller network mesh size (i.e., higher entanglement density, smaller empty voids) prevents the filaments from moving as quickly together as in the actin-microtubule composites.

Finally, the shift that actin-microtubule composites exhibit at ~140 min (in Phase III) from relatively fast contraction (~13 *μ*m/min) to slower contraction of ~3 *μ*m/min (Figure 3) may indicate a shift from contraction triggered by the osmotic-like force from the cations (albeit delayed) to the slow rearrangement of filaments to increase their configurational entropy. We expect that if we delayed the onset of Phase III (lowering of Mg^2+^ concentration) that rapid contraction of actin-microtubule networks would still occur at the same experimental time, governed by the intrinsic relaxation timescale of the network. However, we expect that the shift to slower contraction that occurs in Phase III would occur proportionally later, as it is determined by a shift to configurational entropy maximization that can only occur after the network has been in the presence of low ion concentration for a long enough time. In future work we will more fully explore these hypotheses by varying the times over which the networks are exposed to high and low cation concentrations as well as the maximum Phase II cation concentration.

We chose to focus our study on Mg^2+^ due to its importance in physiological processes such as actin and microtubule polymerization and myosin-driven actin contraction [1, 2, 17, 33, 48]. We expect that other divalent ions, such as Ca^2+^, would produce similar results for polyelectrolytes with similar charge densities as actin and microtubules [23]. However, previous studies examining cation-driven bundling of actin have shown that monovalent ions are not able to induce substantial actin self-association [23, 38]. Further, while Mg^2+^ promotes actin polymerization and stabilizes filamentous actin, Ca^2+^ can destabilize actin filaments and promote depolymerization, which could negatively impact contractile behavior [33].

We have previously shown that actin-microtubule composites exhibit increasing stiffness, mesh size and heterogeneity as the ratio of microtubules to actin is increased [33]. In future work, we will explore the dependence of Mg^2+^-triggered contraction on this ratio. We expect our results to smoothly vary between the two cases we present here as we vary this ratio. While the networks we have studied do not have any crosslinking proteins present, we plan to incorporate actin and microtubule crosslinkers and determine their impact on the results. Based on our previous microrheology measurements on crosslinked actin-microtubule composites [49, 50], we expect to measure a more complicated dependence of crosslinker type and concentration on the contraction behavior.

## CONCLUSION

We have presented an experimental approach that combines diffusion-controlled microfluidics with two-color fluorescence confocal microscopy to measure bulk morphological changes to cytoskeletal networks triggered by increasing and decreasing concentrations of magnesium ions. We show that both actin networks as well as actin-microtubule networks undergo bulk contraction triggered by increasing Mg^2+^ concentration. The contraction dynamics are highly dependent on the network type, with actin-microtubule networks exhibiting a substantially delayed response relative to actin networks. We also show that both networks continue to contract during subsequent lowering of Mg^2+^ concentration, which we attribute to slow network rearrangement to maximize the configurational entropy of the filaments. Our results demonstrate unexpected ways in which the cytoskeleton can dynamically morph and contract in response to environmental stimuli, and how the varying mechanical and structural properties of actin and microtubules can tune the characteristics of this dynamic response.
